# The Book World of Medicine and Science

**Published:** 1899-11-18

**Authors:** 


### THE BOOK WORLD OF MEDICINE AND SCIENCE.

The Radcliffe Infirmary, Oxford: A Practical Scheme for Maintaining the Continuity of Annual Subscriptions during the Lifetime, and for a Number of Years (more or less) after the Subscriber's Decease or Migration, through the Generous and Facile Agency of the Post Office Savings Bank. By E. Vaughan Jenkins. (Oxford: Alden and Co. 1899. Price 2s. 6d.)

A sentence contained in a letter written to the *Times* by Lady Frederick Cavendish, on behalf of a convalescent home, set working in the mind of Mr. Vaughan Jenkins an idea, and that idea led in the course of its development to the production of this little book. The sentence was this: "Heavy extra expense has necessarily been incurred, and, as is the case with many long-established charities, the subscription list dwindles through death, change, and the competition of new undertakings." Falling upon the fertile mind of Mr. Vaughan Jenkins, this simple statement started the idea: "What an excellent thing it would be if a scheme were devised for maintaining the continuity of annual subscriptions during the lifetime and for a number of years, more or less, after the subscriber's decease or migration." Utopian as it seemed, the author, after careful consideration, came to the conclusion that such a thing might be possible if it were worked through the "generous and *facile* agency of the Post Office Savings Bank," and this book is devoted to a complete description of how it could be put in practice. The whole scheme hinges (1) on the capitalisation of the value of an annual subscription on the basis of it being the subscriber's intention to continue it for life, and (2) upon then persuading the subscriber to pay down in advance the capital value of his intended life-long subscription. As one says in regard to clubs and scientific societies, he is to become a "life" instead of an "annual" member. If we accept the author's calculations as correct, a prepayment of twenty-five guineas, deposited in the Post Office Savings Bank on the condition of one guinea being withdrawn by the hospital every year and regarded as the donor's annual subscription, would take about forty-two years to become exhausted, and it is suggested that during this forty-two years the donor if living, or his representatives if dead, shall receive the "recommendations" or "out-patient turns" due for this subscription. The plan is ingenious, and is illustrated in a variety of ways by the author, and we quite agree that if every guinea subscriber could be induced to give twenty-five guineas down, the hospitals would benefit in proportion. But then so they would even if this scheme were not in operation, so that we must regard the scheme rather as a plan for attracting these prepayments than as of any great value in itself. As one of the many modes of raising money we do not feel inclined to offer any criticism of the proposal. Modes of giving must be invented which shall be attractive to all kinds of givers, and although there are many who never will give more than seems necessary at the moment, there are, no doubt, a considerable number who dislike constant appeals and could easily be induced to give a lump sum if some plausible excuse, like that offered by this scheme, were put before them. But when it comes to considering the effect of such a plan upon the management of our great charities we have our doubts. It is not the money alone that a man gives which is of use to a hospital. His constant interest in the institution is of even greater service. The money is sure to come if the interest is there, and so far as management is concerned, the power of withdrawing his subscription is one of the most useful powers which a governor possesses. Life governors are by no means governors of the best sort, and a proposal such as this, which is practically one to create life governorships on a large scale, while no doubt putting the hospitals on a secure footing, might not thereby tend much to their good management. Moreover, any plan which tends, as this one would, to involve a hospital in definite engagements for forty years ahead, is much to be deprecated. So long as life governors are but a small number among a mass of annual subscribers, their "rights" are of no great importance. The general policy of the institution is decided by the annual subscribers. But when life governors are created wholesale, and when these governors are given certain rights in return for money paid, and when, as in this scheme, these rights take forty-two years to run out, it is obvious that for this long period the policy of the hospital in regard to such matters as are affected by these rights is taken out of the hands of those who will be responsible for its active management. Take, as an example, the "recommendations." Nothing is more open to dispute at the present day than the questions of "hospital letters" and of the investigation into the circumstances of applicants and the refusal of the unfit. Yet a hospital which received subscriptions in advance, according to the proposed plan, would be tied down and bound to continue the obnoxious system for a period of forty-two years. Who is to know what will happen in that time?

